# Mechanisms of upper airway muscle control in sleep reveal therapeutic targets for obstructive sleep apnea

**DOI:** 10.1093/ajrcmb/aanag089

**Published:** 2026-06-18

**Authors:** Richard L Horner, D Andrew Wellman, Scott A Sands, Ali Azarbarzin, Luigi Taranto-Montemurro

**Affiliations:** Department of Physiology, University of Toronto, Toronto, ON, Canada; Department of Medicine, University of Toronto, Toronto, ON, Canada; Division of Sleep and Circadian Disorders, Brigham and Women’s Hospital and Harvard Medical School, Boston, MA, United States; Division of Sleep and Circadian Disorders, Brigham and Women’s Hospital and Harvard Medical School, Boston, MA, United States; Division of Sleep and Circadian Disorders, Brigham and Women’s Hospital and Harvard Medical School, Boston, MA, United States; Division of Sleep and Circadian Disorders, Brigham and Women’s Hospital and Harvard Medical School, Boston, MA, United States; Apnimed, Inc., Cambridge, MA, United States

**Keywords:** obstructive sleep apnea, upper airway neuromuscular dysfunction, hypoglossal motor nucleus, aroxybutynin plus atomoxetine, OSA pharmacotherapy

## Abstract

Obstructive sleep apnea (OSA) is the most prevalent sleep-related breathing disorder and is associated with cardiovascular, metabolic, neurocognitive, and mortality risk. OSA arises from recurrent upper airway collapse during sleep, producing intermittent hypoxia and sleep fragmentation. While anatomical vulnerability contributes to airway instability, a key determinant of OSA pathophysiology is the sleep-related reduction in upper airway neuromuscular activity that occurs at the wake–sleep transition. Despite this central role, no approved pharmacologic therapies have targeted the neuromuscular mechanisms underlying airway collapse. This review summarizes the biological basis of upper airway neuromuscular dysfunction in OSA, integrating insights from preclinical models of hypoglossal motor control with clinical evidence supporting neuromodulatory treatment strategies. We focus on AD109, an investigational oral therapy combining a norepinephrine reuptake inhibitor (atomoxetine) with an antimuscarinic agent (aroxybutynin), designed to counteract sleep-related withdrawal of excitatory noradrenergic drive and rapid eye movement (REM)–related muscarinic inhibition at the hypoglossal motor nucleus. Early phase clinical studies demonstrated rapid and substantial improvements in airway collapsibility and apnea–hypopnea index, providing proof of concept for this approach. Results from large phase 3 trials confirm that targeting neuromuscular dysfunction can produce reductions in airway obstruction and meaningful improvements in oxygenation, including hypoxic burden, a metric closely linked to OSA-related sequelae. Symptomatic patients also experienced improvements in fatigue, sleepiness, and snoring versus placebo. Together, these findings support neuromuscular dysfunction as a tractable therapeutic target in OSA and highlight the potential of pharmacologic strategies to address both the physiological consequences of intermittent hypoxia and patient-relevant outcomes across a broad and heterogeneous OSA population.

## Graphical abstract

**Graphical Abstract:**
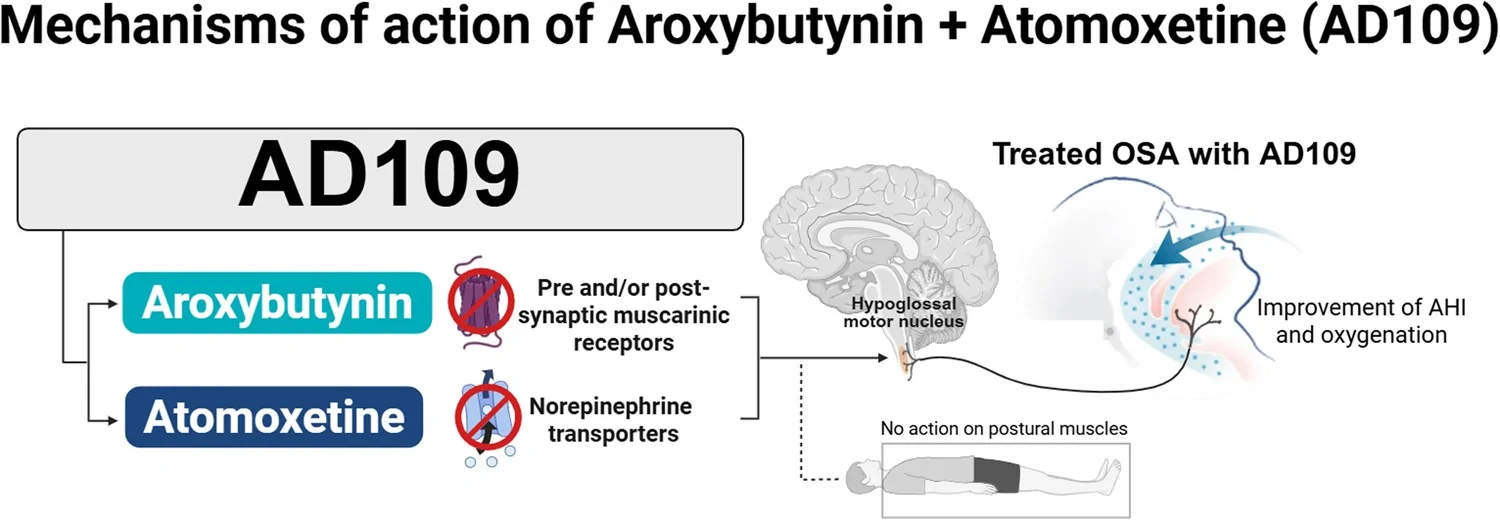

## Introduction

Obstructive sleep apnea (OSA) is the most common sleep-related breathing disorder, affecting individuals across all ages, sexes, races, and weight classes.^1^^,^^2^ OSA is characterized by recurrent upper airway collapse during sleep, which leads to repeated breathing interruptions and drops in oxygen saturation. These events generate OSA-related intermittent hypoxia and disrupt sleep quality. Intermittent hypoxia experienced during sleep is a major driver of OSA sequelae, including cardiovascular and cerebrovascular diseases, metabolic dysfunction, neurocognitive impairment, and increased mortality risk.^3^ Importantly, many patients with OSA experience substantial physiological burden despite having few or no classic symptoms such as excessive daytime sleepiness.^4^ This disconnect contributes both to delays in diagnosis and to the large number of individuals who remain undiagnosed altogether.^5^

A key contributor to OSA is sleep-related reduction in neuromuscular activity. During wakefulness, upper airway dilator muscles receive strong neural input that keeps the airway open.^6^ As individuals transition from wake to sleep, this excitatory drive decreases and leads to pharyngeal collapse in patients with a narrow upper airway.^7^ Thus, OSA typically arises from the interaction of sleep-related neuromuscular relaxation and anatomical vulnerability. The aim of this review is to summarize the key biological mechanisms underlying OSA, with emphasis on neuromuscular dysfunction as a central and modifiable contributor. We also discuss how targeting sleep-related neuromuscular activity with AD109, a novel investigational pharmacologic therapy, may reduce airway obstruction, improve oxygenation, and mitigate the downstream effects of intermittent hypoxia.

## Epidemiology and unmet needs

OSA affects an estimated one billion people globally and up to 80 million adults in the United States.^1^^,^^2^ While OSA was originally described as a disease affecting middle-aged, sleepy men with obesity, recent epidemiological studies have shown broad clinical heterogeneity across the full range of demographic groups and weight classes. Indeed, nonobese adults, women, and younger individuals are frequently affected, challenging traditional stereotypes about OSA.^8-10^ Patients with OSA experience physiologic stress,^11^ leading to fatigue, sleepiness, impaired concentration, increased accident risk,^12^ and long-term cardiovascular, neurocognitive, and oncologic sequelae.^13-16^ Current therapies provide benefits but leave important gaps. Positive airway pressure therapy is effective when used consistently, yet many patients struggle with acceptance or long-term adherence.^17^ Weight-loss interventions^18^ and hypoglossal nerve stimulation^19^ benefit selected subgroups but have important limitations. Notably, no approved pharmacologic treatment directly addresses the sleep-related loss of upper airway muscle tone that contributes to airway collapse in virtually all patients with OSA. Therapies that restore upper airway neuromuscular activity may improve outcomes across the heterogeneous OSA population.

## Upper airway neuromuscular dysfunction as a key target for pharmacological treatment

Current drug development in OSA reflects the understanding that OSA arises from a combination of anatomical and nonanatomical contributors.^20^ The first US Food and Drug Administration–approved pharmacotherapy for OSA, the glucagon-like peptide-1 (GLP-1)/glucose-dependent insulinotropic polypeptide (GIP) receptor agonist tirzepatide, primarily targets the upper airway anatomy in obese patients by reducing upper airway crowding through weight loss.^18^ However, anatomic narrowing is not the only factor causing OSA. Nonanatomical causes contribute to OSA in approximately 70% of patients. These include ventilatory control instability (high loop gain), a low respiratory arousal threshold, and poor pharyngeal muscle activation during sleep, each contributing to recurrent collapse in distinct ways.^21^ High loop gain reflects instability of ventilatory control, in which small changes in carbon dioxide provoke exaggerated oscillations in breathing that promote airway collapse.^22^ A low arousal threshold causes premature awakenings that fragment sleep and prevent effective recruitment of upper airway muscles, perpetuating obstruction. Among the nonanatomical causes of OSA, however, neuromuscular activation represents an important therapeutic target because upper airway patency during both wakefulness and sleep depends on adequate tone of pharyngeal muscles, which compensate for the absence of rigid structural support in the human pharynx.^23^

During wakefulness, individuals with OSA typically compensate for narrowed airway anatomy through increased baseline upper airway dilator muscle activity, maintaining patency despite structural vulnerability.^6^ Therefore, at sleep onset, in patients with OSA the physiological reduction in upper airway muscle tone is larger^7^ compared to that in normal subjects and precipitates airway collapse. Research shows that patients with OSA experience not only a larger decline in muscle activation but also an impaired ability to augment dilator activity in response to the negative pressure generated by upper airway obstruction, highlighting a deficit in neuromuscular responsiveness.^24-26^ When combined with an anatomic predisposition to airway narrowing, this drop in muscle tone leads to airway collapse and obstructed breathing.

## Preclinical studies and mechanistic findings

**Figure 1:**
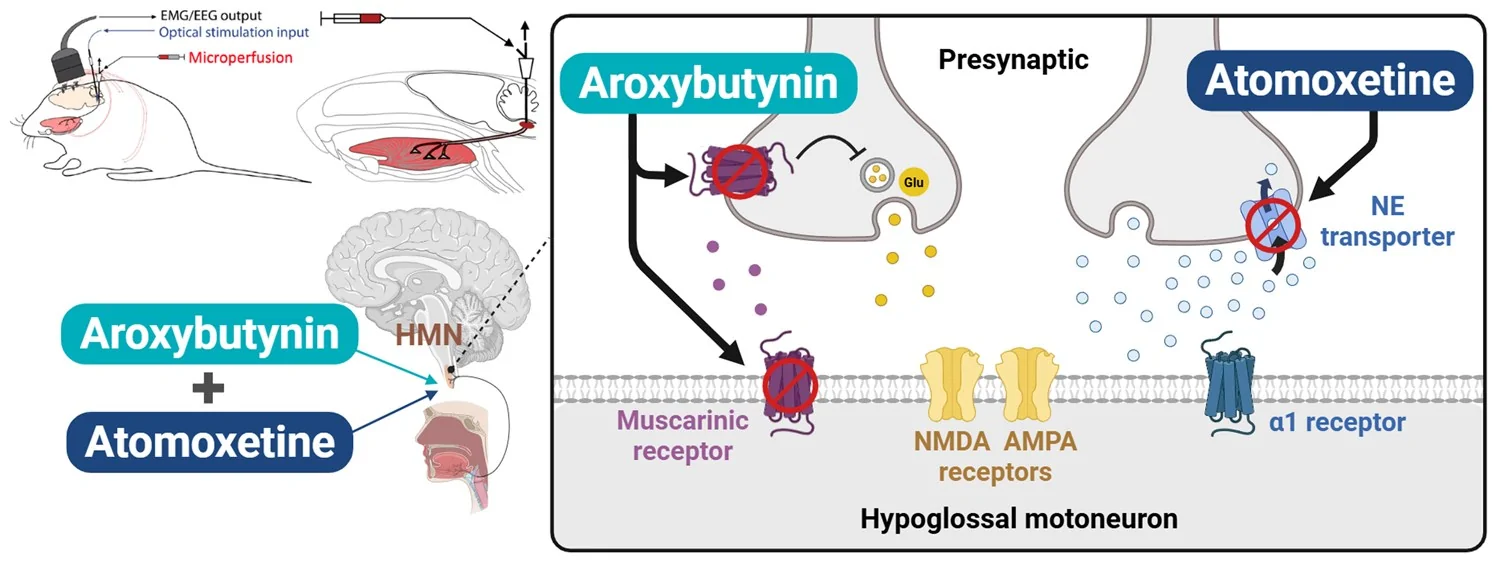
Schema showing mechanisms of control of the hypoglossal motor nucleus (HMN) and the proposed impacts of atomoxetine and aroxybutynin. Presynaptic inputs to the HMN modulate motor output and responsiveness via release of neurochemicals that act on excitatory and inhibitory pre- and/or postsynaptic receptors. This schema shows how increased synaptic norepinephrine (NE) produced by atomoxetine inhibition of the NE transporter would lead to increased α1 receptor–mediated excitation of the HMN. A second important source of HMN excitation is via glutamate acting on non-NMDA and AMPA receptors (ie, non-*N*-methyl-d-aspartate and α-amino-3-hydroxy-5-methyl-4-isoxazolepropionic acid, respectively). In this context, presynaptic muscarinic receptors inhibit glutamate neurotransmission of respiratory drive. As such, aroxybutynin could augment HMN activity and responsiveness via blocking inhibitory pre- and/or postsynaptic muscarinic receptors. In vitro and in vivo animal studies support this schema, including those with chronic recordings of sleep, tongue, and diaphragm motor activities from electroencephalogram (EEG) and electromyogram (EMG) electrodes along with simultaneous pharmacological, optical, or respiratory (eg, hypercapnic) manipulation of the HMN and respiratory drive. See “Mechanism of motor disfacilitation” and “Mechanism of motor inhibition” for further details.

Motoneurons constitute the final common output pathway for the influence of the brain on motor behavior.^27^ For this reason, significant attention in respiratory physiology has focused on identifying mechanisms of control of the hypoglossal motor nucleus (HMN) that innervates the tongue musculature that is considered critical to OSA pathogenesis.^28^ Preclinical research from multiple laboratories have contributed to this body of research using various experimental models. The models span in vitro slice preparations containing the HMN and the core neuronal machinery generating respiratory output,^29-32^ to anesthetized or decerebrate in vivo animal preparations including foundational work using a pharmacologically induced “REM [rapid eye movement] sleep-like state,”^33-35^ to freely behaving in vivo animal preparations with local pharmacological manipulations of the HMN across natural sleep–wake states (Figure 1).^23^^,^^36-38^ Together, these and other models and contributions have led to a framework of the major control mechanisms operating at the HMN across natural sleep–wake states, and thus provide potential rational physiological targets for modulation and translation to OSA pharmacotherapy.^20^^,^^39^^,^^40^

### Mechanisms of motor disfacilitation

Disfacilitation—that is, withdrawal of excitatory input—is a major mechanism of HMN control from wakefulness to sleep.^33^^,^^34^^,^^37^^,^^38^ Noradrenergic, serotonergic, and glutamatergic sleep-sensitive drives contribute to this suppression of HMN activity in both natural sleep^41-44^ and the pharmacologically induced REM sleep-like state.^34^^,^^45^ The extent to which each of these drives are responsible for the loss of pharyngeal motor activity in sleep is now understood to be contingent on important factors such as experimental preparation (eg, natural or pharmacologically induced REM sleep, vagotomized or vagus nerve intact), airway anatomy (eg, compromised upper airways in bulldogs), or level of respiratory drive (eg, hypercapnia).^38^ These factors led to an initial overemphasis of the role of serotonin from earlier studies.^38^ Nevertheless, one dominant observation spanning this research across preparations was that reduced noradrenergic excitation acting on α1 receptors at the HMN contributes to decreased motor activity in sleep.^34^^,^^38^^,^^41^^,^^45^

This endogenous noradrenergic excitation at the HMN is present in wakefulness, reduced in non-REM (NREM) sleep, and further reduced in REM sleep.^34^^,^^38^^,^^41^^,^^45^ It is these findings that led to the notion that noradrenergic reuptake inhibition could provide a pharmacological strategy to restore endogenous noradrenergic drive and offset its natural withdrawal in NREM sleep and its further reduction in REM sleep.^39^^,^^46-52^ Nevertheless, provision of noradrenergic stimulation at the HMN may not be sufficient *by itself* to increase pharyngeal motor tone as REM sleep mechanisms strongly suppress the motor activation responses to a variety of excitatory inputs and neurotransmitters at the HMN, including α1 receptor stimulation and hypercapnic respiratory stimulation.^38^^,^^41^^,^^53-55^ These foundational observations and responses to noradrenergic and respiratory stimulation at the HMN suggested that strong inhibitory mechanisms were being recruited in REM sleep, but at the time these were not identified.

### Mechanisms of motor inhibition

**Figure 2:**
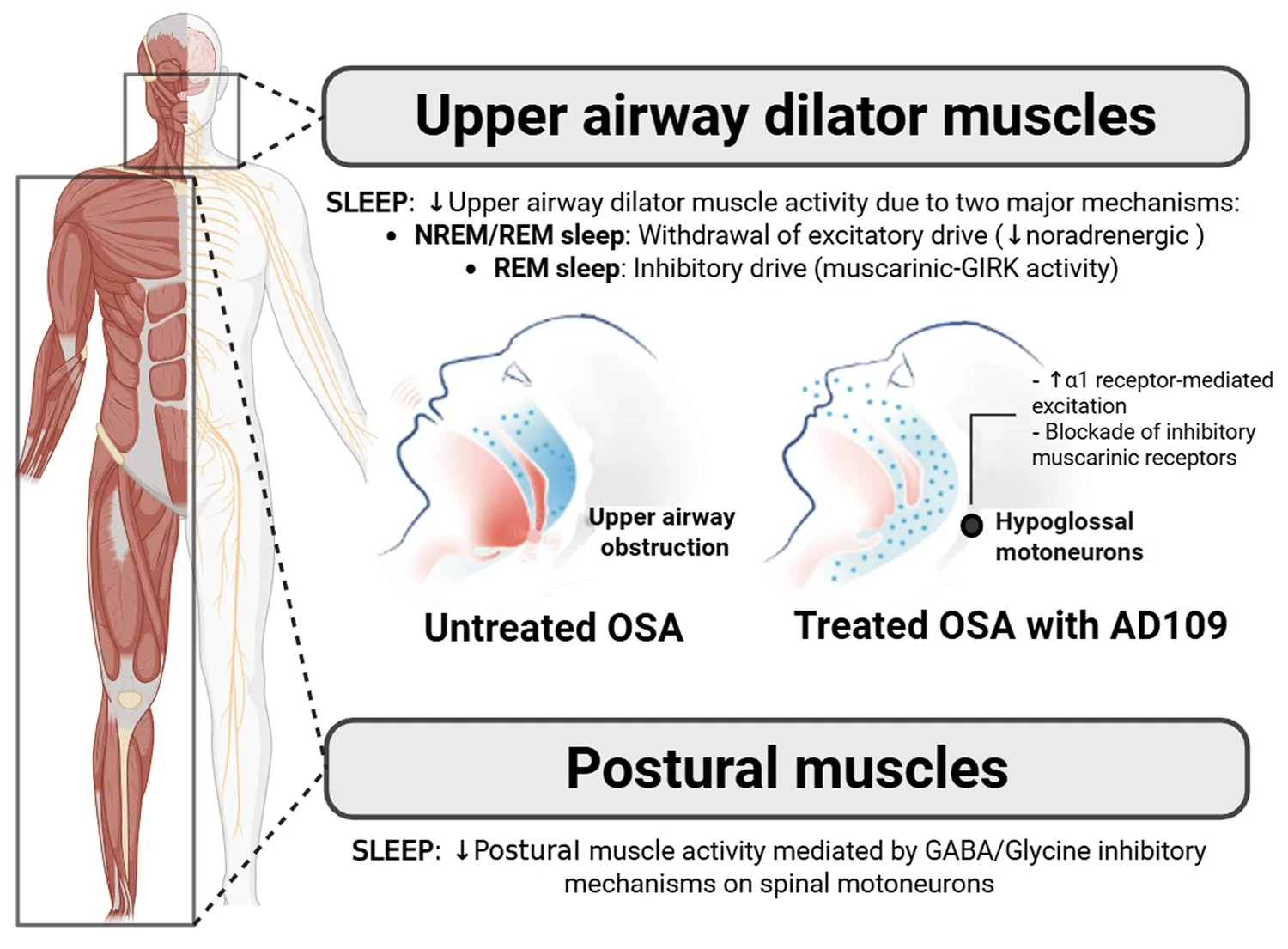
Upper airway dilator muscle activity decreases during sleep due to withdrawal of excitatory drive, including noradrenergic input, in rapid eye movement (REM) and non-REM (NREM) sleep, and recruitment of inhibitory muscarinic–GIRK (G-protein–coupled inwardly rectifying potassium) mechanisms in REM sleep at upper airway motor nuclei. These changes promote airway collapse in obstructive sleep apnea (OSA). In contrast, postural muscle atonia during REM sleep is primarily mediated by GABAergic and glycinergic inhibition at spinal motoneurons. AD109 combines norepinephrine reuptake inhibition (atomoxetine) to restore excitatory drive and antimuscarinic activity (aroxybutynin) to reduce inhibition, thereby stabilizing upper airway muscle tone and improving airway patency.

Inhibition of postural muscle tone is a defining feature of REM sleep, and for spinal motoneurons this is mediated by γ-aminobutyric acid (GABA) and glycine.^56^^,^^57^ Some hypoglossal motoneurons appear to receive some of this inhibition type in REM sleep.^58-61^ However, for overall HMN motor output and pharyngeal motor tone, inhibitory GABA and glycine mechanisms contributed minimally to motor suppression in REM sleep.^62-66^ Most notably, blockade of this putative mechanism at the HMN and trigeminal motor pools had trivial and weakest effects in REM sleep.^36^^,^^37^^,^^62-66^ A different mechanism had to be operating (Figure 2).

Subsequent studies identified that a muscarinic receptor mechanism functionally linked to G-protein–coupled inwardly rectifying potassium (GIRK) channels operates at the HMN.^67^ Blockade of this pathway revealed that its largest inhibitory influence was in REM sleep with lesser or no effects in waking or NREM sleep—that is, a pattern of response expected of a mechanism that is recruited or most strongly influential in REM sleep.^36^^,^^37^^,^^67^

It was largely based on these preclinical mechanisms of pharyngeal motor control in sleep that antimuscarinic agents in combination with noradrenergic reuptake inhibitors were initially tested for OSA pharmacotherapy (see, eg, ^46-50^^,^^52^^,^^68^^,^^69^). Further mechanistic studies are needed to reduce the knowledge gap between preclinical and clinical findings and to link the basic mechanisms identified in animal models to the effects observed in human investigation and clinical studies. Specifically, a more detailed understanding of the source and operation of the cholinergic modulation of HMN activity across sleep–wake states would provide a better understanding of the beneficial responses to systemic administration of OSA pharmacotherapies.^46-50^^,^^52^

## Mechanism of action of atomoxetine and aroxybutynin (AD109)

### Clinical proofs of concept and phase 2 studies

As introduced above, preclinical models indicated that sleep-related loss of noradrenergic activity and increased muscarinic inhibition were primarily responsible for the loss of upper airway muscle tone in sleep, and thus may be key modifiable contributors to upper airway collapse at sleep onset in anatomically susceptible individuals.

AD109 was developed based on the hypothesis that combining 2 complementary pharmacologic actions could counter these mechanisms. Atomoxetine, a selective norepinephrine reuptake inhibitor, was expected to increase synaptic norepinephrine and enhance central excitatory drive to upper airway motor neurons, thereby improving dilator muscle tone. Aroxybutynin, a selective antimuscarinic agent, was hypothesized to reduce muscarinic inhibition of HMN activity (Figures 1 and 2). Together, this dual mechanism was designed to stabilize upper airway muscle activation across NREM and REM sleep and mitigate sleep-related relaxation of the airway.

Early clinical studies established proof of concept for this neuromodulatory strategy. The initial randomized crossover trials using atomoxetine plus oxybutynin showed substantial improvements in OSA severity and upper airway physiology, including reductions in collapsibility and improved muscle responsiveness to negative pressure.^48^^,^^49^ Subsequent studies evaluating other combinations of noradrenergic and antimuscarinic agents further supported this approach, confirming the original finding with variable effect sizes across compounds and study designs.^47^^,^^52^^,^^68^^,^^69^ Oxybutynin was also found to attenuate a reduction in arousal threshold observed with atomoxetine monotherapy, raising the possibility that blocking muscarinic signaling blunts the behavioral/arousal expression of noradrenergic drive^48^ allowing for HMN activations while maintaining sleep.

AD109 refined the original combination by substituting oxybutynin with aroxybutynin, the R-enantiomer that mediates most of oxybutynin’s antimuscarinic actions.^70^ Concentrating the pharmacologic activity in a single active stereoisomer allows for equivalent effects on the upper airway at a lower total dose, with the potential for fewer off-target effects and better overall tolerability. Clinical data with the atomoxetine–aroxybutynin combination confirmed the earlier proof of concept in phase 2 studies. One study evaluated 2 doses of AD109 containing different amounts of atomoxetine (aroxybutynin 2.5 mg combined with either 37.5 mg or 75 mg of atomoxetine) in a randomized, single-night, crossover design in 32 adults with mild to moderate OSA (apnea–hypopnea index [AHI] 5-20 events/hour).^71^ Both AD109 doses produced statistically significant and clinically meaningful reductions in AHI after just one night of treatment. Median AHI decreased from 13.2 (interquartile range [IQR], 8.0-19.1) events/hour on placebo to 5.5 (IQR, 2.2-9.6) events/hour with the higher AD109 dose (*P* <.001) and to 7.8 (IQR, 4.0-13.7) events/hour with the lower dose (*P* <.05), demonstrating rapid onset of action.

Subsequently, the MARIPOSA study, a phase 2b trial, tested 2 doses of AD109 containing different doses of aroxybutynin (atomoxetine 75 mg combined with either 2.5 mg or 5 mg of aroxybutynin) versus atomoxetine alone and placebo in 211 adults with AHI between 10 and 45 events/hour over 4 weeks of treatment.^50^ Compared to placebo, AD109 at the dose of 2.5/75 mg reduced AHI by 47%, from a median (IQR) of 20.5 (12.3-27.2) to 10.8 (5.6-18.5) events/hour (on-treatment estimand, *P* <.0001). The increased dose regimen of aroxybutynin (5/75 mg) did not deliver further improvement. Atomoxetine monotherapy produced smaller AHI reductions and disrupted sleep. AD109 at the dose of 2.5/75 mg also improved self-reported fatigue compared to placebo and atomoxetine monotherapy when measured using the Patient-Reported Outcome Measurement Information System (PROMIS) questionnaire.

AD109 was generally well tolerated, with mostly mild and transient adverse events. These results provided a strong foundation for evaluating AD109 in the large phase 3 SynAIRgy and LunAIRo trials, designed to test whether targeting neuromuscular dysfunction can offer a scalable, nightly oral therapy for a broad OSA population.

### Phase 3 trials

#### Efficacy

SynAIRgy and LunAIRo were the 2 largest phase 3 trials ever performed for an OSA pharmacotherapy and included 1306 adult subjects treated for up to 6 months (SynAIRgy, *N* = 646) or 1 year (LunAIRo, *N* = 660) with AD109 at the aroxybutynin/atomoxetine doses of 2.5/75 mg.^72^ Patients enrolled were adults with an AHI >5 who refused or did not tolerate continuous positive airway pressure (CPAP) and had at least mild fatigue symptoms (PROMIS Fatigue 8a T-score >50.4). Patients in all body mass index (BMI) classes, from normal weight up to BMI 42 kg/m^2^ for women and up to 40 kg/m^2^ for men, and of all OSA severities, from mild to severe, were enrolled in the trials. The patients’ characteristics matched those of patients in sleep clinics who reject CPAP, with approximately equal numbers of males and females and higher proportion of patients with mild to moderate OSA compared to severe OSA. In both trials, AD109 showed statistically significant improvements in both upper airway obstruction and oxygenation,^73^^,^^74^ confirming, like previous trials, the therapeutic relevance of targeting sleep-related neuromuscular dysfunction.

The SynAIRgy study, recently published in *American Journal of Respiratory and Critical Care Medicine*,^75^ evaluated the efficacy of AD109 in a population composed of 35% mild, 42% moderate, and 23% severe OSA. In the intent-to-treat (ITT) estimand (including all randomized participants regardless of treatment adherence or discontinuation), at week 26, the placebo-adjusted least-squares mean difference in AHI was −4.0 events/hour (*P* = .001), whereas in the on-treatment estimand (including data collected while participants remained on assigned therapy), the treatment difference was −6.5 events/hour (*P* <.0001). Median AHI decreased from 19.8 to 13.3 events/hour in the AD109 group in ITT set (compared with 19.1 to 17.7 events/hour with placebo), corresponding to a model-based reduction of 44.1% from baseline (vs 17.6% with placebo; *P* <.0001). In the on-treatment estimand, median AHI decreased from 19.3 to 10.6 events/hour with AD109, corresponding to a model-based 55.6% reduction from baseline (vs 19.1% with placebo; *P* <.0001). Thus, in both analysis sets, the observed median AHI decreased from the moderate to the mild OSA severity category. The magnitude and pattern of AHI reduction in SynAIRgy were similar to those observed previously in the MARIPOSA phase 2 study.

While these results demonstrate statistically significant reductions in AHI, the magnitude of change should be interpreted in the context of a population predominantly composed of patients with mild to moderate OSA. In this setting, clinical benefit may be better captured by relative reductions in AHI, responder analyses, and shifts in disease severity. Accordingly, a greater proportion of participants receiving AD109 achieved clinically meaningful reductions in AHI compared to placebo, including 70% to 80% reduction, and disease control (AHI <5 events/hour) was achieved in approximately one-quarter of the treated patients. These findings highlight the heterogeneity of treatment response and suggest that mean changes alone may underestimate the clinical benefit in subsets of patients. More details about response in different subgroups are present in the SynAIRgy manuscript,^75^ and further insights are expected from future pooled analyses.

AD109 also improved measures reflecting the physiological burden of nocturnal hypoxemia, including hypoxic burden (HB) and oxygen desaturation index (ODI). HB captures both the depth and duration of oxygen desaturation and is calculated as the integrated area under the oxygen desaturation curve associated with respiratory events over sleep time. It has been more closely linked to cardiovascular and neurocognitive sequelae than AHI.^15^ Therefore, the magnitude of improvement in HB observed with AD109 (44.7% vs 8.5% on placebo in ITT and 60.5% vs 14.7% while on treatment) represents a physiologically meaningful change that may have the potential to translate into reductions in cardiovascular risk, although this has not been directly assessed. Improvements in oxygenation were observed across sleep stages, consistent with enhanced upper airway stability throughout the night.

While not all participants reported prominent symptoms, those who were more symptomatic at baseline (Epworth Sleepiness Scale ≥10) experienced statistically significant improvements in sleepiness and fatigue assessed through the PROMIS questionnaires (Fatigue and Sleep Impairment) with AD109 compared to placebo. In those considered asymptomatic at baseline, evidence of symptomatic benefit was not detected.

AD109 also reduced snoring intensity and duration, a disruptive expression of upper airway vibration and obstruction. This finding is consistent with the intervention’s action of raising airway muscle tone to maintain airway patency during sleep.

#### Safety and tolerability

Across phase 3 trials, no deaths or treatment-related serious adverse events were observed with AD109. The overall adverse event profile was consistent with the known pharmacology of its individual components (noradrenergic reuptake inhibitors and antimuscarinic agents), which have been used clinically for several decades. No unexpected safety signals were identified.

As expected, not all participants tolerated AD109. Despite the above improvements in OSA severity, discontinuations due to adverse events occurred in 21.3% of AD109-treated participants versus 3.1% on placebo at week 26. Most discontinuations due to adverse events happened in the first weeks of the trials and the main reasons were insomnia (4.9%), urinary hesitation (2.5%), and nausea (2.2%). Otherwise, adverse events were typically mild and transitory and included dry mouth, insomnia, nausea, and urinary hesitation. The adverse event profile is consistent with the mechanism of action of AD109. Noradrenergic stimulation may contribute to wake-promoting effects, including insomnia and nausea, while antimuscarinic activity may explain anticholinergic symptoms such as urinary hesitancy and dry mouth.

As expected based on the known pharmacology of norepinephrine reuptake inhibition, treatment was associated with small increases in blood pressure (1.4 mm Hg vs placebo) and heart rate (3.6 beats per minute vs placebo). According to the American College of Cardiology, in a 55-year-old man with hypertension, a 2 mm Hg increase in systolic blood pressure is associated with a minimal change in 10-year coronary vascular disease risk (approximately from 4.3% to 4.4%).^76^ However, while these changes were small on average, their clinical relevance may vary depending on individual patient characteristics, and careful consideration may be warranted in patients with uncontrolled or resistant hypertension, heart failure, or tachyarrhythmias. Notably, such patients were excluded from the clinical trials.

AD109 was associated with a reduction in REM sleep, consistent with the established effects of noradrenergic, serotonergic, and tryciclic antidepressant agents. The clinical significance of pharmacologically driven REM reduction remains uncertain, and existing evidence suggests that it is not associated with impairments in cognitive performance or vigilance.^50^^,^^77^ However, the possibility that some individuals may experience clinical consequences related to REM sleep reduction, particularly over longer-term exposure, cannot be excluded.

Several factors may have contributed to tolerability challenges. Enrollment was limited to individuals who had refused or were unable to tolerate CPAP, potentially enriching the study population for patients with low arousal threshold^78^ or disturbed sleep phenotype,^79^ traits that have been linked to both CPAP intolerance and greater sensitivity to the wake-promoting properties of atomoxetine.^80^ This remains a hypothesis that warrants further evaluation, including studies assessing whether dose adjustment or patient selection based on these characteristics may improve tolerability. Tolerability may also have been influenced by interindividual variability in atomoxetine metabolism related to cytochrome P450 2D6 (CYP2D6) status. Individuals with reduced CYP2D6 activity may have higher systemic exposure to atomoxetine, which could contribute to variability in adverse events such as insomnia as well as in heart rate and blood pressure responses. While early data indicate that a lower dose of AD109 could have meaningful efficacy, particularly in patients with milder disease,^71^ dose adjustment was not permitted in the SynAIRgy or LunAIRo trials, and future studies incorporating dose-flexible approaches may help optimize tolerability while preserving efficacy.

## Importance of OSA treatment and potential contribution of AD109

OSA is typically treated for 2 primary reasons: (1) to improve daytime symptoms and functional impairments in individuals who report excessive sleepiness, fatigue, or other OSA-related symptoms; and (2) to reduce long-term cardiovascular disease (CVD) risk, mainly in those who are asymptomatic. OSA is strongly associated with increased CVD risk through multiple mechanisms. However, across animal and human studies, OSA-related hypoxemia has emerged as a key culprit linking OSA to adverse cardiovascular outcomes. In patients with OSA, recurrent upper airway obstruction leads to repeated oxygen desaturations, creating a pattern of chronic intermittent hypoxia (CIH), a phenomenon well characterized in animal models and associated with profound systemic effects. CIH is characterized by repetitive cycles of hypoxia and reoxygenation, which induce oxidative stress, increase production of reactive oxygen species, and trigger endothelial dysfunction and systemic inflammation.^81^ These processes promote atherosclerosis,^82^ impair vascular reactivity,^83^ and activate platelets,^84^ providing a mechanistic explanation for the strong association between OSA and CVD. In parallel, nightly exposure to OSA-related hypoxia activates the sympathetic nervous system in a cyclic pattern leading to surges in heart rate, and in turn blood pressure (30-40 mm Hg), that often lead to persistent elevations in blood pressure during wakefulness.^85^ CIH also disrupts metabolic pathways, contributing to insulin resistance, glucose intolerance,^86^ dyslipidemia,^87^ and weight gain,^88^ creating a bidirectional relationship between metabolic dysfunction and OSA.

The systemic effects of OSA-related intermittent hypoxia and sleep fragmentation translate into a broad spectrum of clinical sequelae. Numerous cohort studies demonstrate that OSA is associated with increased risk of hypertension, coronary artery disease, heart failure,^89^ atrial fibrillation, stroke,^90^ and cardiovascular mortality.^91^ Importantly, large epidemiologic data show that measures of OSA-related hypoxia, particularly HB, are stronger predictors of cardiovascular outcomes than AHI^15^ and treatment benefits,^92^^,^^93^ underscoring the severity of nocturnal hypoxemia as a central determinant of long-term CVD risk.

CIH also has profound effects on the brain. Recurrent hypoxia and sleep fragmentation impair memory consolidation, attention, and executive function.^94^^,^^95^ Neuroimaging studies reveal structural and functional changes in the hippocampus, prefrontal cortex, and white matter pathways in untreated OSA.^96-98^ Clinical studies report that treating OSA reduce cognitive impairment, dementia,^99^ and Parkinson’s disease,^100^ suggesting that OSA contributes to neurodegenerative processes. Emerging evidence indicates an association between OSA and cancer incidence and progression.^13^^,^^14^

While some patients experience prominent symptoms such as excessive daytime sleepiness or fatigue, a substantial proportion of individuals with OSA are minimally symptomatic despite carrying significant physiological burden.^4^^,^^9^^,^^101^ In patients with daytime symptoms, short-term consequences of OSA, including impaired alertness,^102^ decreased work productivity,^103^ and higher rates of motor vehicle^104^ and occupational accidents,^12^ add to the urgency of effective therapies.

By enhancing upper airway muscle activity during sleep, AD109 directly targets the neuromuscular dysfunction that leads to airway collapse. In multiple clinical studies, AD109 produced consistent reductions in AHI over several months, decreased the proportion of patients with OSA or reduced disease severity class (ie, from severe to moderate or mild), and improved key oxygenation measures including HB, ODI, and percentage of total sleep time with oxygen saturation <90%, showing clear improvement of parameters related to CIH. Because the HB of OSA is strongly associated with cardiovascular, neurocognitive, and metabolic risk,^105^ these improvements have the potential to reduce the long- and short-term consequences of OSA.

Beyond the effects on upper airway obstruction, among patients who were more symptomatic at baseline, AD109 led to improvements in subjective outcomes compared to placebo, including reductions in daytime fatigue and sleepiness.

Taken together, these findings indicate that AD109 has the potential to provide a pharmacologic approach addressing the core pathophysiology of OSA and supporting treatment goals across a broad range of patients.

## Future directions

OSA is a multifactorial disease driven by a combination of anatomical vulnerability and several nonanatomical traits, including sleep-related neuromuscular dysfunction, ventilatory control instability, and variations in arousal threshold. These physiological traits interact to determine the presence and severity of OSA in each individual patient. Future progress of OSA pharmacotherapy will depend on the development of precision, multimodal treatment strategies that target multiple pathophysiological traits simultaneously.

One important direction involves therapies that address the anatomical component of airway collapsibility. In obese patients, significant weight loss can reduce pharyngeal tissue volume and improve upper airway patency. As mentioned above, GLP-1/GIP receptor agonists such as tirzepatide have demonstrated clinically meaningful reductions in AHI in individuals with obesity-related OSA.^18^ These agents may complement neuromuscular therapies by reducing mechanical load on the upper airway.

Another emerging approach involves targeting ventilatory control instability (loop gain). Elevated loop gain contributes to oscillatory breathing and can amplify the propensity for obstructive events. Sulthiame, a carbonic anhydrase inhibitor, has shown the ability to reduce loop gain and stabilize breathing,^106^ with meaningful AHI reductions over 3 months of treatment.^107^ By acting on ventilatory control rather than anatomy or muscle tone, sulthiame offers a distinct mechanistic pathway that may be valuable in combination regimens.

Together, these advances open the possibility of pathophysiology-directed combination therapy. AD109, which targets the neuromuscular deficit responsible for sleep-related upper airway relaxation, could be paired with agents such as tirzepatide or sulthiame to address additional anatomical or ventilatory control contributors within a dose-flexible and potentially endotype-driven approach. Such combinations may provide additive or synergistic benefits by simultaneously improving airway structure, stabilizing airway muscle activation, and correcting ventilatory control abnormalities. As more therapeutic options emerge, the field is expected to move beyond a one-treatment-fits-all paradigm and toward personalized, mechanism-based treatment strategies that expand the number of patients who can achieve durable control of OSA.

## Supplementary Material

aanag089_Supplementary_Data
